# Associations of objectively measured moderate-to-vigorous physical activity and sedentary behavior with quality of life and psychological well-being in prostate cancer survivors

**DOI:** 10.1007/s10552-016-0787-5

**Published:** 2016-07-28

**Authors:** Cadeyrn J. Gaskin, Melinda Craike, Mohammadreza Mohebbi, Jo Salmon, Kerry S. Courneya, Suzanne Broadbent, Patricia M. Livingston

**Affiliations:** 1 Deakin University, Faculty of Health, Locked Bag 20001, Geelong, VIC 3220 Australia; 2 Institute of Sport, Exercise and Active Living, College of Sport and Exercise Science, Victoria University, Melbourne, Australia; 3 Deakin University, Faculty of Health, Biostatistics Unit,, Geelong, Australia; 4 Deakin University, School of Exercise and Nutrition Sciences, Geelong, Australia; 5 Behavioural Medicine Laboratory, Faculty of Physical Education and Recreation, University of Alberta, Edmonton, AB Canada; 6 School of Health and Human Sciences, Southern Cross University, Lismore, NSW Australia

**Keywords:** Accelerometer, Exercise, Physical activity, Sedentary behavior, Sedentary time, Prostate cancer

## Abstract

**Purpose:**

Although evidence is building on the positive effects of physical activity for prostate cancer survivors, less is known about the possible independent effects of sedentary behavior on quality of life and psychological well-being in this population. We determined the extent to which objectively measured moderate-to-vigorous physical activity (MVPA) and sedentary behavior were independently associated with quality of life, anxiety, and depressive symptoms in prostate cancer survivors.

**Methods:**

An exploratory cross-sectional analysis was undertaken on baseline data from a multicenter, cluster randomized controlled trial on the efficacy of a clinician referral and 12-week exercise program for men who had completed active treatment for prostate cancer. Multiple regression analyses were performed using data from 98 prostate cancer survivors who wore hip-mounted accelerometers (time spent sedentary defined as <100 counts per minute [CPM]; MVPA defined as >1,951 CPM) and completed self-report instruments on their quality of life, anxiety, and depressive symptoms. Results were compared with minimal clinically important differences for the quality of life scales.

**Results:**

Independent of sedentary behavior, increases in MVPA of between 15 and 33 min/day were associated with clinically important (but not statistically significant) improvements in three quality of life scales (insomnia, diarrhea, and financial difficulties). Independent of MVPA, decreases in sedentary behavior of 119 and 107 min/day were associated with clinically important (but not statistically significant) improvements in physical functioning and role functioning, respectively.

**Conclusion:**

Within our exploratory study, modest increases in MVPA and more substantive decreases in sedentary behavior were independently associated with clinically important improvements in several quality of life scales. Further research, including prospective studies, is required to understand sedentary behavior across larger and more representative samples (in terms of their physical, psychological, and social functioning and their engagement in physical activity) of prostate cancer survivors.

**Trial registration:**

Australia and New Zealand Clinical Trials Register (ANZCTR): ACTRN12610000609055

## Introduction

Prostate cancer is the second most common cancer in men worldwide and has a high survival rate [1]. Having been diagnosed with prostate cancer, however, is associated with poorer mental health (e.g., increased anxiety, depressive symptoms, and psychological distress) [2, 3], functional limitations (e.g., urinary, bowel, and sexual dysfunction) [4], low levels of moderate-to-vigorous physical activity (MVPA) [5, 6], and reduced quality of life [2, 4]. Engaging in physical activity can ameliorate many of the adverse effects of prostate cancer and its treatments, with systematic review findings strongest for the positive effect of physical activity on aerobic endurance, muscular endurance, and quality of life in this population [7, 8]. Conversely, evidence is emerging that sedentary behavior poses a health risk that is independent of insufficient physical activity [9–11]. Sedentary behavior is defined as “any waking behavior characterized by an energy expenditure ≤1.5 METs [metabolic equivalents] while in a sitting or reclining posture” [12]. From research with adults, there is strong evidence that sedentary behavior is associated with all-cause mortality, fatal and non-fatal cardiovascular disease, type 2 diabetes, and metabolic syndrome independent of physical activity [13]. Comparisons between men with and without history of prostate cancer, however, have yielded inconsistent findings with respect to time spent sedentary [14, 15], and few studies have focused on the association between sedentary behavior and health outcomes (particularly mental health outcomes) in prostate cancer survivors [5, 16]. Greater understanding of the independent effects of physical activity and sedentary behavior on the quality of life and psychological well-being of prostate cancer survivors is warranted and would assist in the design of targeted interventions to improve the lives of prostate cancer survivors.

The ENGAGE (efficacy of a referral and physical activity program for survivors of prostate cancer) study was a multicenter, cluster randomized controlled trial to determine the efficacy of a clinician referral and 12-week exercise program to increase physical activity among men who had completed active treatment for prostate cancer [17, 18]. Compared to men in the control condition, those in the intervention significantly increased their vigorous physical activity levels and experienced increased cognitive functioning and reduced depressive symptoms [18]. This trial is one of only a few studies involving prostate cancer survivors that included measures of quality of life and psychological well-being, as well as objective measures of physical activity and sedentary behavior. Given the potential utility inherent in understanding these relationships for the development and refinement of interventions to improve health outcomes, we conducted a secondary analysis of the ENGAGE study baseline data. The aim of this exploratory cross-sectional analysis was to determine the extent to which MVPA and sedentary behavior were independently associated with quality of life, anxiety, and depressive symptoms in prostate cancer survivors prior to commencement of the exercise program. We also assessed whether the associations found could be clinically important.

## Methods

### Study population

The ENGAGE study recruitment and sample details have been described previously [17, 18]. Inclusion criteria were men diagnosed with stage I, II, or III prostate cancer who had (a) completed active treatment for prostate cancer within the previous 3–12 months (patients on hormone treatment were eligible to participate), and (b) the ability to complete surveys in the English language. Patients were excluded if they had any musculoskeletal, cardiovascular, or neurological disorders that could limit them from exercising. Eligible patients were recruited through the outpatient clinics of three large public health services and four private clinics located in metropolitan Melbourne, Australia. The patients’ treating clinicians provided medical clearance for all participants prior to their involvement in the exercise program. Of the 741 patients screened for this study, 443 met the eligibility criteria, and 147 were contactable and agreed to participate. Of these 147 participants, 98 provided complete accelerometer, quality of life, anxiety, and depressive symptoms data (34 chose not to wear accelerometers, 13 provided invalid accelerometer data, and 2 had incomplete quality of life, anxiety, and depressive symptoms data).

Ethics approval to conduct this study was obtained from the human research ethics committees of the health services and host university involved in this study. Informed consent was obtained from all individual participants included in the study.

### Measurements

Baseline data on demographics, clinical characteristics, quality of life, anxiety, depressive symptoms, physical activity, and time spent sedentary were collected. Demographics and clinical characteristics obtained through self-report questionnaires included: age, height, weight, relationship status, highest level of education, and treatment regime. Self-reported heights and weights were used to calculate body mass index (BMI) (kg/m^2^) scores. Clinical characteristics obtained from medical records included: stage of disease, weeks since active treatment, and health service type (public/private).

Quality of life was measured using the European Organization for Research and Treatment of Cancer core quality of life questionnaire (EORTC QLQ-C30_(V3)_) [19] and the prostate tumor-specific module (EORTC QLQ-PR25) [20]. The EORTC QLQ-C30_(V3)_ has a global health status scale, five functional scales (physical, role, cognitive, emotional, and social), and nine symptom scales (fatigue, nausea and vomiting, pain, dyspnea, insomnia, appetite loss, constipation, diarrhea, and financial difficulties). The EORTC QLQ-PR25 has two functional scales (sexual activity and sexual functioning) and four symptom scales (urinary symptoms, bowel symptoms, hormonal treatment-related symptoms, and incontinence aid). Both measures have convergent and discriminant validity, as well as adequate internal consistency reliability [19, 20]. Minimal clinically important differences for the scales (each of which ranges from 0 to 100) have been estimated to be approximately 5–10 points [21–23]. This estimation strongly overlaps with guidelines for small-sized, clinically relevant differences (ranging from 3 to 7 points for diarrhea to 6–19 points for role functioning) produced from a method combining a systematic review, a meta-analysis, and expert opinions [24].

Anxiety was measured with the Memorial Anxiety Scale for Prostate Cancer (MAX-PC) [25]. The MAX-PC has three subscales (prostate cancer anxiety, prostate-specific antigen anxiety, and fear of recurrence) and a total anxiety scale. The MAX-PC has concurrent validity with established anxiety measures (e.g., the anxiety subscale of the Hospital Anxiety and Depression Scale [26]), discriminant validity, internal consistency, and test–retest reliability [25, 27].

Depressive symptoms were measured using the Centre for Epidemiological Studies Depression Inventory (CES-D) [28]. The scale has strong concurrent validity with both clinical and self-report criteria, and sound construct validity [28].

Physical activity and time spent sedentary were measured using hip-mounted ActiGraph GT1 M (Pensacola, FL) units. The accelerometer is a valid and reliable tool for measuring physical activity and sedentary time among adults [29–31]. Each participant was shown how to wear the accelerometer on a nylon belt over the right hip (physical activity estimates do not vary by right or left hip placement [32]) and was also provided with written instructions on the use of the accelerometer. Participants were asked to start wearing the accelerometer when they got out of bed the next morning and asked to wear it for seven consecutive days during waking hours. On completion of the seven days, participants were asked to return the accelerometer in a reply-paid envelope. Data from the ActiGraph units were processed using ActiLife software (V6.7.1) and managed using a customized Microsoft Excel macro. Time spent sedentary was defined as <100 counts per minute [CPM], and MVPA was defined as >1,951 CPM [30]. To be included in the analysis, participants were required to have worn the accelerometer for at least 10 h each day (60 min or more of consecutive zero counts, without “tolerance,” was considered non-wear of the device) for at least four of the seven days (based on Healy et al. [31]). Average daily minutes in MVPA and time spent sedentary were calculated for each participant based on the number of valid days of data provided. Due to differences in daily accelerometer wear time between participants, sedentary behavior was standardized to a 12-hour wear time using the formula:

$$\frac{\text{time spent sedentary}}{\text{accelerometer wear time}} \times 60\,{\text{min}}\times 12\,{\text{h}}$$

This standardized sedentary behavior variable was used in the analyses.

Current guidelines suggest that prostate cancer survivors engage in at least 150 min/week of moderate-intensity physical activity or 75 min/week of vigorous-intensity physical activity or an equivalent combination of moderate and vigorous physical activity, which may include weight-bearing exercises [33].

### Statistical analysis

Statistical analyses were performed using Stata (Version 13) software. Independent *t* tests and Chi-squared tests were used to determine whether there were differences between men with and without complete data in terms of their demographic and clinical characteristics. Subsequent analyses were performed on the data from the men with complete data.

The sexual functioning and incontinence aid scales were omitted from the analysis due to a high amount of non-responses (62.2 and 70.4 %, respectively). Responding to these items was conditional on participants being sexually active in the last 4 weeks and wearing incontinence aids, respectively. For the remaining variables, a negligible amount of data were missing (<0.01 %). The result from Little’s [34] test (*χ*^2^(390) = 399.21, *p* = .36) suggests that data were missing completely at random.

Using multiple regression analyses, MVPA and standardized sedentary behavior (as continuous variables) were simultaneously regressed against each of the quality of life, anxiety, and depressive symptoms scales and subscales. Adjusted multiple regression analyses were also performed with demographic variables (age, BMI, relationship status, and highest level of education), clinical variables (number of comorbidities, weeks since active treatment, stage of disease, treatment regime, and health service type), and clinician (to assess the effect of clustering) screened as potential covariates or factors. Variables that were related (*p* ≤ .10) to any of the quality of life, anxiety, and depressive symptoms scales and subscales in bivariate analyses were included in the initial adjusted regression models. Using backward elimination, covariates and factors were then removed from these models (one by one, in an iterative process) when *p* ≥ .05. Because these analyses focused on estimation, rather than prediction, attention is paid to the unstandardized beta coefficients and their confidence intervals (rather than effect sizes for the proportions of variance explained). The unstandardized beta coefficients represent the changes in quality of life, anxiety, and depressive symptom scores per 1 min/day increase in MVPA or standardized sedentary behavior after adjusting for the other covariates and factors included in each model.

Exact *p* values are reported together with unstandardized beta coefficients and their confidence intervals. Although adjusting the α value to protect against inflation of experiment-wise error when multiple tests are performed is generally advisable [35, 36], making such an adjustment in this study could result in Type II errors, which may discourage researchers from further investigations in this area. Given that our prime focus was on hypothesis generation, rather than hypothesis validation, we did not adjust for multiple comparisons. Accordingly, *α* was set at 0.05. Sample size calculations for the ENGAGE study were based on the primary outcomes for the main trial [17, 18], rather than the exploratory secondary analyses reported here. Modest recruitment and the constraints of fixed-term funding meant that the target of recruiting 220 participants [17] was not achieved.

Given that the study was exploratory, we also assessed whether the associations could be clinically important, irrespective of their statistical significance. A minimal clinical important difference can be defined as “the smallest difference in score in the domain of interest which patients perceive as beneficial and which would mandate, in the absence of troublesome side effects and excessive cost, a change in the patient’s management” [37]. The results from the multiple regression analyses were compared with minimal clinically important differences for the quality of life scales using two methods. First, the changes in MVPA and, separately, in sedentary behavior needed to obtain minimal clinically important differences were calculated. Minimal clinically important differences in quality of life scales were defined as the lower limits of the ranges for small-sized, clinically relevant differences provided in published guidelines [24]. A small-sized difference is one that is subtle, but clinically relevant. For each scale, the changes in MVPA per day, and (separately) in sedentary behavior per day, needed to obtain clinically important differences were calculated by dividing the small-sized clinically important difference by the adjusted unstandardized beta coefficients. Second, the changes in quality of life scores from performing recommended levels of MVPA were determined. Prostate cancer survivors are recommended to undertake at least 150 min/week of moderate physical activity or 75 min/week vigorous physical activity or an equivalent combination of moderate and vigorous physical activity [33, 38, 39]. For the purpose of this analysis, this guideline was translated into a daily recommendation of 21 min (150 min/7 days) of MVPA. For each scale, the effect of performing recommended levels of MVPA was calculated by multiplying the daily recommendation for MVPA (21 min/day) and the adjusted unstandardized coefficient. Using published guidelines, the resulting change in quality of life score was interpreted as being trivial (no difference or unlikely to have clinical relevance), small (subtle, but nevertheless clinically relevant), medium (likely to be clinically relevant), or large (unequivocal clinical relevance) [24]. The analysis did not include the anxiety or depressive symptoms scales, because, as far as we are aware, clinically important differences for these scales have yet to be established.

## Results

Participant demographic and clinical characteristics are provided in Table 1. Briefly, the men had a mean age of 65.6 years (SD = 8.5), were overweight (BMI: *M* = 28.0, SD = 3.7), and had last undergone active treatment, on average, 25.3 weeks prior (SD = 10.0). Compared to men who did not have complete data (*n* = 49), those with complete data (*n* = 98) were, on average, 5 years older (*p* < . 01) and had undergone different treatment regimes (i.e., more likely to have been treated with both surgery and radiotherapy and less likely to have undergone surgery only; *p* = .04) (see Table 1). There were no statistically significant differences for BMI, relationship status, highest level of education, number of comorbidities, weeks since active treatment, stage of disease, and health service type. Participants wore the accelerometers, on average, 14 h/day (SD = 1.4) for between 4 and 8 days (*M* = 6.5, SD = 0.9). They spent, on average, 38 min/day (SD = 22) engaged in MVPA and 10 h/day (SD = 1.5) in sedentary behavior (standardized for accelerometer wear time to 9 h/day, SD = 0.8).

**Table 1:** Comparison of demographic and clinical characteristics of men with and without complete data

Characteristics	Complete data available		Effect size	*p*
	Yes (*n* = 98)	No (*n* = 49)		
*Demographic characteristics*				
Age, *M* (SD) years	67.3 (8.0)	62.1 (8.6)	*d* = 0.62	<.01
Body mass index, *M (*SD*)* kg/m^2^	27.9 (3.7)	28.3 (3.6)	*d* = −0.10	.57
Relationship status			*V* = 0.10	.21
Married/partnered, *n* (%)	83 (84.7)	35 (76.1)		
Separated/divorced/widowed/single, *n* (%)	15 (15.3)	11 (23.9)		
Highest level of education			*V* = 0.14	.23
Primary/secondary school, *n* (%)	33 (34.0)	22 (47.8)		
Certificate or diploma, *n* (%)	37 (38.1)	12 (26.1)		
University degree, *n* (%)	27 (27.8)	12 (26.1)		
*Clinical characteristics*				
Number of comorbidities, *M* (SD)	1.8 (1.5)	1.9 (1.3)	*d* = −0.06	.76
Weeks since active treatment, *M* (SD)	26.1 (10.1)	23.7 (9.7)	*d* = 0.24	.17
Stage of disease			*V* = 0.07	.77
Stage I, *n* (%)	33 (38.8)	15 (40.5)		
Stage II, *n* (%)	36 (42.4)	17 (45.9)		
Stage III, *n* (%)	16 (18.8)	5 (13.5)		
Treatment regime			*V* = 0.24	.04
Surgery only, *n* (%)	37 (37.8)	27 (55.1)		
Radiotherapy only, *n* (%)	14 (14.3)	7 (14.3)		
Surgery and radiotherapy, *n* (%)	27 (27.6)	4 (8.2)		
ADT with surgery and/or radiotherapy, *n* (%)	20 (20.4)	11 (22.4)		
Health service type			*V* = 0.07	.43
Public, *n* (%)	74 (75.5)	34 (69.4)		
Private, *n* (%)	24 (24.5)	15 (30.6)		

*d* effect size for independent *t* tests; *V* effect size for Chi-squared tests; *ADT* androgen deprivation therapy

In general, unstandardized beta coefficients for associations between MVPA and quality of life, anxiety, and depressive symptoms scales and subscales were larger than those between sedentary behavior and these variables (Table 2). None of the analyses returned statistically significant results, however.

**Table 2:** Descriptive statistics and regression analyses involving quality of life, anxiety, and depressive symptoms, and their relationships with both moderate-to-vigorous physical activity and sedentary behavior (mins/day)

	Descriptive statistics		Unadjusted analyses				Adjusted analyses				Covariates/factors
			MVPA		SB		MVPA		SB		
	*M*	*SD*	B (95 % CI)	*p*	B (95 % CI)	*p*	B (95 % CI)	*p*	B (95 % CI)	*p*	
*Quality of life*—*global health status scale* ^a^											
Global health status	77.38	15.69	0.05 (−0.13, 0.23)	.57	−0.03 (−0.11, 0.05)	.48	0.05 (−0.13, 0.23)	.57	−0.03 (−0.11, 0.05)	.48	
*Quality of life*—*functional scales* ^a^											
Physical	92.11	10.62	0.10 (−0.01, 0.22)	.08	−0.03 (−0.08, 0.02)	.23	0.06 (−0.07, 0.20)	.33	−0.04 (−0.10, 0.01)	.14	^g^
Role	90.82	20.18	0.01 (−0.22, 0.24)	.95	−0.06 (−0.16, 0.05)	.28	0.01 (−0.22, 0.24)	.95	−0.06 (−0.16, 0.05)	.28	
Cognitive	84.01	17.74	0.02 (−0.18, 0.22)	.85	−0.02 (−0.11, 0.08)	.75	0.02 (−0.18, 0.22)	.85	−0.02 (−0.11, 0.08)	.75	
Emotional	85.37	17.05	−0.00 (−0.20, 0.19)	.98	−0.06 (−0.15, 0.03)	.17	−0.00 (−0.20, 0.19)	.98	−0.06 (−0.15, 0.03)	.17	
Social	86.90	19.33	0.11 (−0.11, 0.33)	.33	0.00 (−0.10, 0.10)	.96	0.06 (−0.16, 0.29)	.57	−0.01 (−0.11, 0.10)	.90	^h^
*Quality of life*—*symptom scales* ^a^											
Fatigue	18.20	15.83	−0.14 (−0.31, 0.03)	.11	0.06 (−0.02, 0.13)	.15	−0.10 (−0.29, 0.10)	.33	0.04 (−0.05, 0.12)	.37	^g i^
Nausea and vomiting	0.68	3.31	0.03 (−0.01, 0.06)	.19	0.01 (−0.01, 0.03)	.24	0.03 (−0.01, 0.06)	.19	0.01 (−0.01, 0.03)	.24	
Pain	13.27	19.14	−0.17 (−0.38, 0.05)	.13	−0.05 (−0.15, 0.05)	.32	−0.07 (−0.29, 0.15)	.53	−0.06 (−0.15, 0.03)	.18	^j^
Dyspnea	10.54	20.08	−0.15 (−0.37, 0.08)	.20	0.07 (−0.03, 0.17)	.15	−0.05 (−0.28, 0.17)	.64	0.06 (−0.04, 0.16)	.22	^j^
Insomnia	22.45	26.55	−0.23 (−0.53, 0.07)	.13	0.01 (−0.13, 0.14)	.91	−0.26 (−0.55, 0.03)	.08	−0.05 (−0.19, 0.09)	.48	^k^
Appetite loss	3.40	11.21	0.01 (−0.12, 0.14)	.91	0.03 (−0.03, 0.09)	.30	0.01 (−0.12, 0.14)	.91	0.03 (−0.03, 0.09)	.30	
Constipation	8.25	14.46	0.04 (−0.12, 0.21)	.60	0.02 (−0.05, 0.10)	.54	0.04 (−0.12, 0.21)	.60	0.02 (−0.05, 0.10)	.54	
Diarrhea	5.56	15.80	−0.09 (−0.27, 0.09)	.32	−0.03 (−0.11, 0.05)	.49	−0.09 (−0.27, 0.09)	.32	−0.03 (−0.11, 0.05)	.49	
Financial difficulties	13.06	23.35	−0.10 (−0.37, 0.17)	.47	0.00 (−0.12, 0.12)	.99	−0.17 (−0.44, 0.10)	.21	−0.04 (−0.17, 0.08)	.48	^h^
*Quality of life*—*prostate cancer functional scales* ^a^											
Sexual activity	30.93	27.22	−0.11 (−0.20, 0.43)	.47	−0.01 (−0.15, 0.13)	.88	−0.02 (−0.33, 0.29)	.89	−0.05 (−0.19, 0.08)	.45	^l^
*Quality of life*—*prostate cancer symptom scales* ^a^											
Urinary symptoms	17.63	15.13	−0.06 (−0.23, 0.12)	.52	0.02 (−0.06, 0.10)	.62	−0.04 (−0.21, 0.13)	.66	0.01 (−0.07, 0.09)	.75	^i^
Bowel symptoms	5.73	9.50	−0.04 (−0.15, 0.07)	.47	0.01 (−0.04, 0.06)	.69	−0.02 (−0.13, 0.09)	.70	−0.01 (−0.06, 0.04)	.73	^k m^
Hormonal treatment-related symptoms	11.85	13.61	−0.09 (−0.24, 0.06)	.23	0.05 (−0.01, 0.12)	.11	−0.03 (−0.14, 0.09)	.63	0.01 (−0.05, 0.06)	.84	^i k m^
*Anxiety*											
Prostate cancer anxiety^b^	6.23	6.50	−0.01 (−0.09, 0.06)	.77	0.01 (−0.03, 0.04)	.78	−0.03 (−0.10, 0.05)	.51	−0.01 (−0.04, 0.03)	.72	^i l^
Prostate-specific antigen anxiety^c^	0.23	0.73	0.00 (−0.01, 0.01)	.96	0.00 (−0.00, 0.00)	.96	−0.00 (−0.01, 0.01)	.47	−0.00 (−0.01, 0.00)	.59	^i^
Fear of recurrence^d^	3.78	3.19	−0.02 (−0.05, 0.02)	.40	−0.01 (−0.02, 0.01)	.44	−0.02 (−0.05, 0.02)	.40	−0.01 (−0.02, 0.01)	.44	
Total anxiety^e^	10.24	8.61	−0.03 (−0.13, 0.07)	.59	−0.00 (−0.05, 0.04)	.94	−0.05 (−0.16, 0.05)	.29	−0.01 (−0.05, 0.03)	.64	^l^
*Depressive symptoms*											
Depressive symptoms^f^	7.95	7.50	−0.00 (−0.09, 0.08)	.96	0.01 (−0.03, 0.05)	.70	0.01 (−0.08, 0.09)	.90	−0.00 (−0.04, 0.04)	.97	^h i^

*MVPA* moderate-to-vigorous physical activity; *SB* sedentary behavior; *B* unstandardized beta coefficient (representing the difference in quality of life, anxiety, and depressive symptom scores per additional 1 min/day of MVPA or SB after adjusting for other covariates and factors included in each model); *CI* confidence interval

Each row in the table includes two multiple regression analysis (one unadjusted and the other adjusted)

^a^Scale range 0–100; higher scores indicative of higher global health status, functioning, and symptoms/problems

^b^Scale range 0–33; higher scores indicative of greater prostate cancer anxiety

^c^Scale range 0–12; higher scores indicative of greater prostate-specific antigen anxiety

^d^Scale range: 0–9; higher scores indicative of greater fear of recurrence

^e^Scale range 0–54; higher scores indicative of greater total anxiety

^f^Scale range 0–60; higher scores indicative of more depressive symptoms

Adjusted for ^g^stage of disease, ^h^clinician, ^i^highest level of education, ^j^number of comorbidities, ^k^body mass index, ^l^age, ^m^treatment regime

Interpretation of the unstandardized beta coefficients with reference to guidelines for minimal clinically important differences in quality of life scores revealed that an increase in MVPA of less than 1 hour per day was associated with clinically important (but not statistically significant) differences in several symptom scales (fatigue, insomnia, diarrhea, and financial difficulties; Table 3). Undertaking recommended levels of MVPA (i.e., 21 min/day, equivalent to 150 min/week) was only related (but not to a statistically significant level) to reductions in insomnia and financial difficulties to a clinically important extent.

**Table 3:** A comparison of the associations of moderate-to-vigorous physical activity and sedentary behavior with minimal clinically important differences in quality of life

	Minimal clinically important differences (points)^a^	Changes in moderate-to-vigorous physical activity needed to attain minimal clinically important differences (mins/day)^b^	Changes in sedentary behavior needed to attain minimal clinically important differences (mins/day)^b^	Changes in quality of life from adhering to recommended guidelines for physical activity (points)^c^	Clinical relevance of the changes in quality of life from adhering to recommended guidelines for physical activity^d^
*Quality of life*—*global health status scale* ^e^					
Global health status	4	77	−138	1.09	Trivial
*Quality of life*—*functional scales* ^e^					
Physical	5	78	−119	1.34	Trivial
Role	6	857	−107	0.15	Trivial
Cognitive	3	150	−200	0.42	Trivial
Emotional	–^f^	–	–	–	–
Social	5	78	−714	1.34	Trivial
*Quality of life*—*symptom scales* ^e^					
Fatigue	5	−52 ^g^	132^i^	−2.02	Trivial
Nausea and vomiting	3	120 ^h^	300^i^	0.53	Trivial
Pain	6	−88 ^g^	−97^j^	−1.43	Trivial
Dyspnea	4	−74 ^g^	67^i^	−1.13	Trivial
Insomnia	4	−15 ^g^	−80^j^	−5.48	Small
Appetite loss	5	714 ^h^	167^i^	0.15	Trivial
Constipation	5	114 ^h^	217^i^	0.92	Trivial
Diarrhea	3	−33 ^g^	−107^j^	−1.91	Trivial
Financial difficulties	3	−17 ^g^	−70^i^	−3.63	Small

^a^Minimal clinically important differences represent the lower limits of the ranges for small-sized, clinically relevant differences provided in published guidelines [24]

^b^Values obtained from dividing the minimal clinically important differences by the adjusted unstandardized beta coefficients from Table 2

^c^Values obtained from multiplying the recommended guidelines for moderate-to-vigorous physical activity (21 min/day) and the adjusted unstandardized beta coefficients from Table 2

^d^Extent of clinical relevance: trivial (no difference or unlikely to have clinical relevance), small (subtle, but nevertheless clinically relevant), medium (likely to be clinically relevant), and large (unequivocal clinical relevance) [24]

^e^Scale range: 0–100; higher scores indicative of higher global health status, functioning, and symptoms/problems

^f^No difference is available for emotional functioning due to difficulties in producing a guideline for this scale [24]

^g^Negative values indicate that increases in moderate-to-vigorous physical activity are associated with less symptoms/problems and decreases in moderate-to-vigorous physical activity are associated with more symptoms/problems

^h^Positive values indicate that increases in moderate-to-vigorous physical activity are associated with more symptoms/problems

^i^Positive values indicate that increases in sedentary behavior are associated with more symptoms/problems

^j^Negative values indicate that decreases in sedentary behavior are associated with more symptoms/problems and increases in sedentary behavior are associated with less symptoms/problems

## Discussion

Within our small study population of prostate cancer survivors, achievable increases in MVPA and reductions in sedentary behavior were associated with clinically important improvements in several aspects of quality of life. These findings support evidence from randomized controlled trials (synthesized in a recent systematic review [40]) showing a positive relationship between physical activity and quality of life in prostate cancer survivors. Our work extends current knowledge through (1) demonstrating the potential independent benefits of increasing MVPA and reducing sedentary behavior for improving quality of life, (2) providing evidence of such relationships when activity levels are objectively measured, and (3) interpreting these effects with reference to minimal clinically important differences.

The magnitudes of the adjusted unstandardized beta weights for the associations between both MVPA and sedentary behavior and both physical functioning and fatigue suggest that clinically important changes on these quality of life scales may be achievable for many men with prostate cancer. Although increasing MVPA by 52–78 min/day or reducing sedentary behavior by 119–132 min/day may be beyond many men with prostate cancer, the independence of these effects suggests that more modest increases in MVPA combined with lower reductions in sedentary behavior are likely to produce clinically important changes. These findings are consistent with those of a systematic review on the effect of exercise on the quality of life of adult post-treatment cancer survivors [41]. In this review, the observed mean difference in EORTC QLQ-C30 physical functioning and fatigue scores from baseline to up to 12 weeks were 6.23 points (95 % CI 1.74, 10.72) and −22.45 (95 % CI −50.66, 5.77), respectively. Although this evidence from randomized controlled trials may suggest that engaging in physical activity enhances physical functioning to a modest extent [41], our data are open to reverse causation. That is, it may also be the case that people who have higher levels of physical functioning participate in higher levels of physical activity.

Several quality of life scales were associated with MVPA to a similar or greater extent than physical functioning and fatigue (social functioning, pain, dyspnea, insomnia, diarrhea, and financial difficulties). Systematic review evidence on adult post-treatment cancer survivors suggests that exercise may improve social functioning and sleep disturbance, but may have no effect on pain [41]. For breast and colon cancer patients undergoing adjuvant chemotherapy, however, a recent study has shown that physical activity can reduce nausea and vomiting, and pain [42]. Furthermore, some associations may be more plausibly explained as reverse causations; diarrhea, for example, may be more likely to decrease someone’s involvement in physical activity than an increase in physical activity would be to decrease diarrhea. More work is needed to identify the circumstances in which physical activity can be effective in reducing cancer-related symptoms. Physical activity may be more effective at times when patients are experiencing higher levels of symptoms.

The findings support advice within physical activity guidelines that exceeding the recommended physical activity levels is likely to provide additional benefits [38, 39]. The interpretations of several of the adjusted unstandardized beta coefficients are that clinically important improvements in several quality of life scales could be achieved through engaging in additional MVPA and reducing sedentary behavior. As prostate cancer emerges at a time of life when many men are retired and may have time to undertake more frequent physical activity, promoting programs that increase activity levels and reduce sedentary behavior may be effective in this population.

The adjusted unstandardized beta coefficients for the associations between sedentary behavior and quality of life illustrate the potential importance of reducing sedentary behavior. Reducing sedentary behavior (e.g., through standing more and sitting less) by less than 2 h/day was associated with clinically important improvements in several quality of life scales (physical functioning, role functioning, dyspnea). As such, reducing sedentary behavior meaningfully compliments the effects achievable through increasing MVPA. Our findings are consistent with those of a systematic review in which emerging evidence was presented of higher levels of sedentary behavior being associated with both lower quality of life [43] and greater risk of depression [44] in adults (not cancer survivors). Research with cancer populations is mixed, with some evidence for an association between sedentary behavior and quality of life [45–48], and other studies showing no effects [16, 49].

Limitations of this study include ceiling and floor effects (many data points at the upper and lower limits, respectively, of response scales) for several of the quality of life, anxiety, and depressive symptoms scales (which attenuated the magnitudes of correlations observed), the participants’ high levels of functioning, the small sample size, potential confounding, and the inability of the accelerometers to detect posture. Men in this study had reasonably high quality of life scores, and negligible levels of anxiety and depressive symptoms, thus producing ceiling and floor effects. The men were highly functioning, with the EORTC QLQ-C30_(V3)_ scores, for example, being consistently higher than, but within one standard deviation of, norms for prostate cancer survivors aged 60–69 years [50]. The men were also more physically active than other samples of prostate cancer survivors reported in the literature [5]. Our exclusion of men with musculoskeletal, cardiovascular, or neurological disorders that could limit them from exercising from this study may have been partially responsible for producing this sample of highly functioning men and the limited variation in much of our data. The sample size in this study was small, meaning that the null findings could have been due to low statistical power and that the reported findings may be unstable and will require replication with larger samples. Even so, the use of an objective measure for assessing physical activity is a major strength of this study, because measurement error (which can reduce statistical power [51]) is substantially less with accelerometers compared to physical activity logs and questionnaires [52]. Although the study was undertaken with data from a cluster randomized controlled trial, the study reported here is cross-sectional, meaning that the results are subject to residual confounding as with any observational study. Finally, the hip-worn ActiGraph accelerometer was unable to detect posture, which means that if a participant was standing still and accumulating <100 CPM, this activity would be incorrectly classified as sedentary time resulting in an over-estimation of activity of this intensity [31].

This study is novel in its focus, as it provided a snapshot of physical activity and time spent sedentary in prostate cancer survivors using an objective measure, and their associations with quality of life, anxiety, and depressive symptoms. The findings suggest it may be possible to achieve clinically important improvements in quality of life through increasing MVPA and reducing sedentary behavior. Further research is needed to examine these relationships more closely using objective measures of sitting, such as the thigh-worn activPAL inclinometer [53]. In addition, researchers have an opportunity to build on the work showing, for example, that patterns of sedentary behavior, such as the frequency of interruptions to sustained bouts of sitting, influence health outcomes irrespective of the total volume of sedentary behavior [54]. Future research, including prospective studies, would ideally involve larger and more representative samples of prostate cancer survivors, in terms of their physical, psychological, and social functioning and their engagement in objectively assessed physical activity.
